# MicroRNA Analysis of In Vitro Differentiation of Spermatogonial Stem Cells Using a 3D Human Testis Organoid System

**DOI:** 10.3390/biomedicines12081774

**Published:** 2024-08-06

**Authors:** Adam B. Cohen, Banafsheh Nikmehr, Omar A. Abdelaal, Megan Escott, Stephen J. Walker, Anthony Atala, Hooman Sadri-Ardekani

**Affiliations:** 1 Wake Forest Institute of Regenerative Medicine, Winston-Salem, NC 27101, USA; bnikmehr@cfi.clinic (B.N.); omarelayman88@gmail.com (O.A.A.); megescot@wakehealth.edu (M.E.); swalker@wakehealth.edu (S.J.W.); hsadri@wakehealth.edu (H.S.-A.); 2 Department of Urology, Atrium Health Wake Forest Baptist, Winston-Salem, NC 27157, USA; 3 Carolinas Fertility Institute, Winston-Salem, NC 27103, USA; 4 Department of Urology, Faculty of Medicine, Zagazig University, Zagazig 7120001, Egypt

**Keywords:** microRNA profiling, biochemical processes, spermatogenesis, human testis organoid

## Abstract

Spermatogenesis produces male gametes from spermatogonial stem cells (SSC), beginning at puberty. Modern-day laboratory techniques allow for the long-term culture of SSC and in vitro spermatogenesis. The specific biochemical processes that occur during spermatogenesis remain poorly understood. One particular element of spermatogenesis that has yet to be characterized is the role of microRNAs (miRNA), short, non-transcribed RNAs that act as post-translational regulators of gene activity. In this study, we seek to describe the presence of miRNA in a two-dimensional (2D) SSC culture and a 3D human testis organoid (HTO) system. Testicular cells were isolated from the frozen tissue of three brain-dead subjects, propagated in cultures for four to five weeks, and used to form 3D HTOs. Following organoid formation, differentiation of testicular cells was induced. RNA was isolated from the whole testis tissue (WT) showing in vivo conditions, HTO Day Zero (2D SSC culture), Day 2 HTOs, and Day 23 differentiated HTOs, then analyzed for changes in miRNA expression using the Nanostring nCounter miRNA panel. One hundred ninety-five miRNAs met the criteria for expression in WT, 186 in 2D culture, 190 in Day 2 HTOs, and 187 in differentiated HTOs. One hundred thirty-three miRNAs were common across all conditions, and 41, 17, 6, and 11 miRNAs were unique for WT, 2D culture, Day 2 HTOs, and differentiated HTOs, respectively. Twenty-two miRNAs were similar between WT and differentiated HTOS. We evaluated the miRNA expression profiles of progressively complex stages of testicular cell culture, culminating in a 3D organoid model capable of meiotic differentiation, and compared these to WT. We identified a great variance between the native tissue and the culture system; however, some miRNAs are preserved. These data may provide avenues for deeper understanding of spermatogenesis and the ability to improve this process in the laboratory. Research on miRNA continues to be an essential avenue for understanding human spermatogenesis.

## 1. Introduction

**Figure 1:**
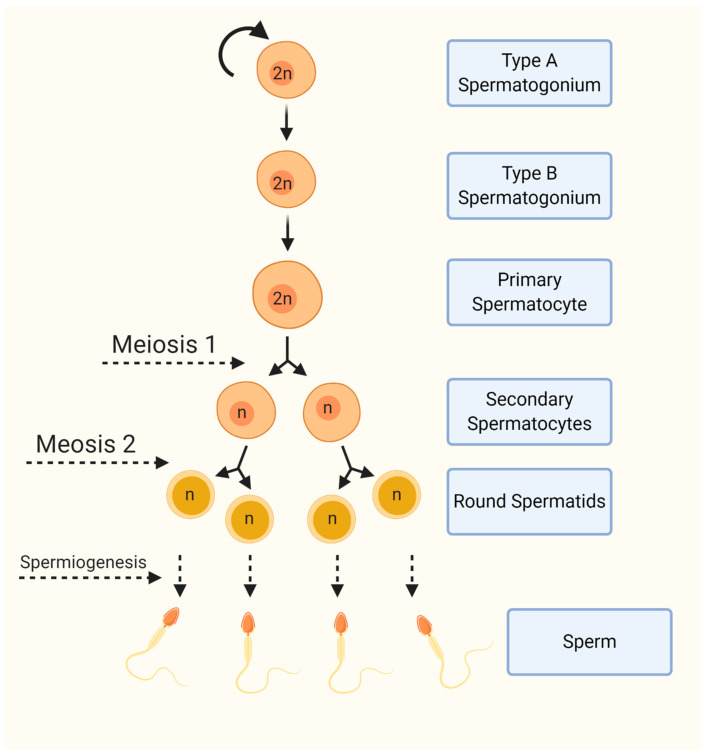
Diagram to illustrate spermatogenesis in the human.

Spermatogenesis, the process through which male gametes (spermatozoa) are formed from germ cells in the seminiferous tubule epithelium in the testis, is a complex process that involves precise hormonal and gene-level control. Within the tubular microenvironment, spermatogonial stem cells (SSCs) located on the basement membrane must undergo successive mitotic divisions to expand the existing pool of germ cells. This pool self-renews through selective division, producing more SSCs and primary spermatocytes, which enter the extended meiotic prophase 1, in which homologous recombination occurs, and eventually divide to create secondary spermatocytes. These cells undergo a crucial meiotic divide to form haploid spermatids, which must further differentiate to take on the mature sperm phenotype through many steps involving morphological change and chromatin condensation [1] (Figure 1). These cells are supported by Sertoli cells, nutrition, and an immunologically privileged niche within the seminiferous epithelium [2]. Hormonal influences tightly control the process of spermatogenesis, relying on regulation from the hypothalamic–pituitary–gonadal axis (HPA). The release of luteinizing hormone (LH) and follicle-stimulating hormone (FSH), which themselves depend on cyclical signaling from the hypothalamus in the form of gonadotropin-releasing hormone, directly influence the release of testosterone from Leydig cells and modulate the actions of Sertoli cells [3]. This hormonal control activates molecular programs specific to each level of differentiating germ cells, guiding the process from SSCs through meiosis and morphological change. The transcriptomes of these developing cells are highly conserved among mammals, designating their importance to reproductive success [4,5].

While most of the human genome is transcribed, only a tiny amount of the resulting RNA is encoded and translated into protein [6,7]. Long thought to be nonsense, the remaining transcripts have been revealed to play extensive roles in gene regulation. Long non-coding RNAs, the prominent examples being H19, XIST, and HOTAIR, are the size of messenger RNAs but often contain no open reading frame and translate to no identifiable protein; still, these molecules play roles ranging from X-inactivation to global gene silencing [8,9,10]. Smaller non-coding RNAs reside in the nucleus and regulate RNA splicing and ribosome biogenesis, and others play active roles in protein translation: the classic ribosomal and transfer RNAs [11]. Of recent importance are small non-coding RNAs (sncRNAs), a class of non-coding RNAs less than 25 nucleotides in length. Within this group are the small interfering RNAs (siRNAs), Piwi-interacting RNAs (piRNAs), and microRNAs (miRNAs) [12], differentiated by length, single or double strand, and biogenesis [13]. miRNAs are double-stranded, often range 17–22 nucleotides in length, and canonically cause post-transcriptional gene regulation through translational repression or destabilization and cleavage of their target mRNAs, of which there can be hundreds [14,15,16].

mRNAs can have several recognition sites for one or many miRNAs [13]. In the genome, miRNAs can be found in intergenic and intragenic regions and are often transcribed by RNA polymerase II as hairpin loop structures [17,18]. They are subsequently processed within the nucleus by a complex of the RNase DROSHA and DGCR8 (DiGeorge Syndrome Critical Region 8), which recognizes the RNA sequence. Following transport out of the nucleus, the primordial miRNAs are processed by Dicer, a cytoplasmic endonuclease, to their mature double-stranded forms, which are unwound and bound to Argonaute proteins to form the RNA-inducing silencing complex (RISC) [19,20]. This complex goes on to recognize target mRNAs and repress their translation.

SncRNAs, including miRNAs, are found abundantly in the testis and have been shown through RT-PCR, microarray, and sequencing studies to have different expression profiles across the constituent cell types located within. These non-coding RNAs have been shown to play a part in spermatogenesis as well as sperm maturation in the epididymis, as evidenced by studies evaluating their role in infertility [21,22]. For example, the 17–92 clusters of miRNAs, following c-Myc activation, repress the translation of E2F1 and prevent apoptosis during meiotic recombination [23]. Other miRNAs, such as miR-181a, have been implicated in the human development of azoospermia through defects in expression of S6K1, overexpression of which led to growth arrest of spermatocytes [24]. Many other studies have revealed roles for miRNAs in spermatogenesis that range from maintaining undifferentiated spermatogonia to facilitating the morphological change associated with sperm maturation [25,26]. Most of these studies used whole testis’ tissue or isolations of specific cell types to reach their conclusions.

**Figure 2:**
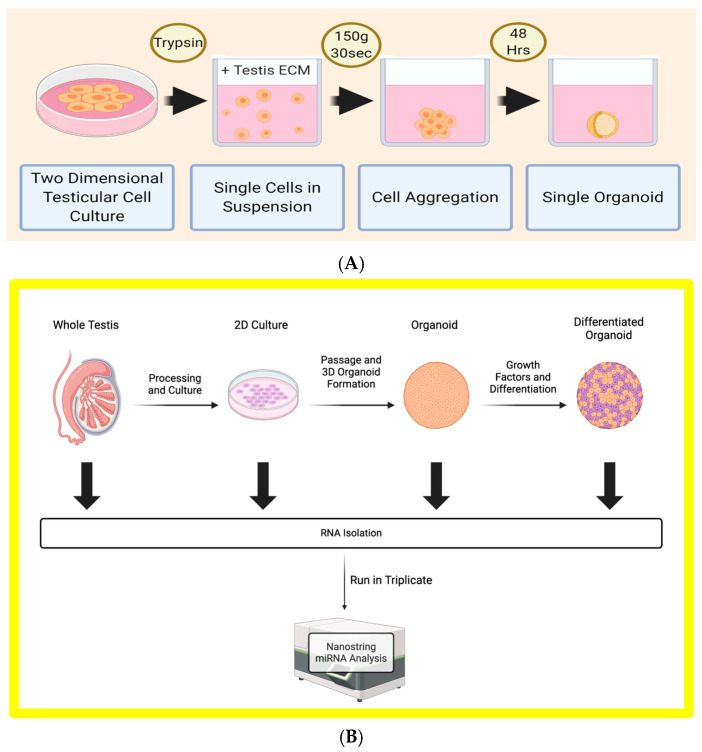
(**A**) Three-dimensional Human Testis Organoid (HTO) formation process from 2D SSC culture. Digestion of propagated adherent testicular cells by Trypsin, addition of human testicular extracellular matrix (ECM), brief centrifugation (150 g/30 s), and 48 h of incubation in 37 °C. (**B**) Experimental design showing comparison of 4 conditions: whole testis (In Vivo), 2D testicular culture, 3D human testis organoid (undifferentiated versus differentiated). This diagram was created with BioRender.com.

Stem cell culture techniques have seen tremendous advances in the past decades, and in 2009, the ability to culture SSCs was demonstrated in human models [27]. At its conception, this technology was intended to expand germ cell numbers for use in SSCs autotransplantation for those receiving gonadotoxic treatments. Sato et al. demonstrated full in vitro spermatogenesis in a murine model [28,29]. Multiple models have been introduced in the past decade to achieve this feat using human material [30,31,32]. Using our own in vitro testis model, a three-dimensional (3D) human testicular organoid (HTO) (Figure 2), we demonstrated the appearance of the canonical germ cell differentiation and post-meiotic markers DAZL, PRM1, and acrosin within HTOs differentiated with retinoic acid, follicle-stimulating hormone (FSH), and stem cell factor (SCF) [33]. To date, we have found no studies evaluating the role of miRNAs and how their expression changes in different stages of testicular cell culture. In this study, we attempt to answer this question by analyzing miRNA expression in the sequential levels of culture: whole testis, established 2D culture, formed 3D HTOs, and differentiated HTOs. With the resulting data, we hope to identify avenues for strengthening the process of in vitro spermatogenesis as well as provide a deeper understanding of this process in native tissue.

## 2. Materials and Methods

### 2.1. Human Materials

Whole testes from three adult brain-dead patients (25, 18, and 56 years old with normal spermatogenesis) were procured through the National Disease Research Interchange (NDRI). These were divided into tiny fragments of tissue (2–5 mm) and cryopreserved in 1x minimal essential medium (MEM) containing 20% fetal bovine serum (FBS) and 8% dimethyl sulfoxide (DMSO), using a rate-controlling Mr. Frosty container (Thermo Fisher Scientific) in a −80 °C freezer overnight. Cryotubes were subsequently moved to liquid nitrogen for long-term storage. Testis decellularization and extracellular matrix (ECM) extraction were performed as previously reported [33].

### 2.2. Testicular Cell Isolation and Culture

Testi tissue was thawed and enzymatically digested to form a cell suspension, then cultured for 4–5 weeks as described previously [27,34]. After 5–6 passages, cells were harvested using 0.25% Trypsin-EDTA (Invitrogen, Carlsbad, CA, USA) to create a cell suspension to form organoids. A portion of this suspension was centrifuged briefly to form a pellet, then snap-frozen in liquid nitrogen for later RNA isolation (Day 0).

### 2.3. Formation of Three-Dimensional Human Testi Organoids (HTOs)

Cell suspensions for organoid formation were centrifuged at 350 G with a brake for 5 min. Then, pellets were re-suspended to a concentration of 1,000,000 cells/mL in organoid formation media: supplemented Stem Pro 34 (Invitrogen) containing 30% FBS, 0.5% penicillin–streptomycin (Invitrogen), 1 µg/mL solubilized human testis extracellular matrix (ECM) (made in our laboratory from normal human testis), 50 µg/mL gentamicin (Thermo Fisher, Waltham, MA, USA), 20 ng/mL recombinant human epidermal growth factor (Sigma-Aldrich, Burlington, MA, USA), 10 ng/mL recombinant human glial cell line-derived neurotrophic factor (Sigma-Aldrich), and 10 ng/mL recombinant human leukemia inhibitory factor (LIF). This cell suspension (at 100 µL/well, 10,000 cells per well) was seeded into 96-well Ultra Low Attachment U-Bottom culture plates (Thermo Fisher), and plates were centrifuged at 150 g for 30 s with brake. Plates were then incubated for 48 h at 37 °C in a humidified atmosphere with 5% CO_2_. Following incubation, 100 µL of media was added to each well, and a portion of the organoids formed from each patient (around 48 HTOs) was harvested, briefly centrifuged to form a pellet, and flash-frozen in liquid nitrogen for RNA isolation (Day 2).

### 2.4. Organoid Differentiation

Following 48 h of incubation, organoid culture medium was replaced with organoid formation media containing no LIF, 2 µM retinoic acid (Sigma-Aldrich), 2.5 × 10^−5^ IU follicle-stimulating hormone (Sigma-Aldrich), and 100 ng/mL recombinant human stem cell factor (Peprotech, Cranbury, NJ, USA), termed “differentiation medium”. Culture media was refreshed every 2–3 days, and organoids were incubated at 34 °C with 5% CO_2_. On day 23 (21 days of culture in differentiation medium), organoids were harvested for additional studies and RNA isolation.

### 2.5. Testicular Cell Culture Characterization

Total RNA, including miRNAs, was isolated from snap-frozen cell pellets collected at the passage of 2D cell culture following 48 h of formation and 21 days of differentiation using the miRNeasy RNA extraction kit (Qiagen, Hilden, Germany). RNA was additionally isolated from snap-frozen whole testis tissue (WT) corresponding to each original cell culture following homogenization using a mortar, pestle, and Qiashredder (Qiagen). RNA concentration and quality were evaluated using a Nanodrop Flourometer (ThermoFisher, Waltham, MA, USA) RNA isolated from whole testis tissue and 2D culture was converted to cDNA using a high-capacity cDNA reverse transcription kit (Life Technologies, Carlsbad, CA, USA). Characterization was performed via real-time qualitative PCR using ABI Taqman primers: THY1, ZBTB16, UCHL1 for undifferentiated spermatogonia; GATA4, SOX9, CLU for Sertoli cells; STAR, TSPO, CYP11A1 for Leydig cells, and ACTA2 for peritubular cells. POLR2A was used as an endogenous control. For each assay, 50 ng of cDNA was used. Cycling conditions were as follows: 95 °C for 10 min, 95 °C for 15 s (40 cycles), and 60 °C for 1 min. All runs were performed in duplicate using whole testis tissue RNA as a control. The expression of all genes was normalized to the POLR2A gene, and relative expression was determined with the ΔΔCT method.

Flow cytometry was further employed to quantify the testicular cell types in the population, principally SSCs. The latter was estimated as the cell population expressing HLA-ABC, CD9, and CD49. BD antibodies were used at a concentration of 5 µL/50,000 cells in 100 µL. This solution was incubated for 1 h at room temperature and then washed with Flow cytometry buffer (1% FBS in PBS). Flow cytometry was performed with a BD Accuri C6 flow cytometry system without sorting, and a total of 10,000 events were recorded per sample.

### 2.6. Organoid Viability Assays

Live-Dead confocal microscopy was performed using the Molecular Probes Live-Dead Cell Imaging kit (Thermo Fisher). Organoids were harvested along with their media, and 2 µL of calcein dye (1:80 in DMSO) and 4 µL of ethidium bromide dye were added and briefly agitated to mix. Then, they were incubated at 37 °C for 30 min. Organoids were imaged on an Olympus FV10i macro-confocal microscope (Olympus, Tokyo, Japan).

### 2.7. miRNA Analysis via Nanostring Sprint Profiler

miRNA analysis was performed in 4 sets of 3 biological replicates using Nanostring nCounter Human v3b miRNA panel, with probes representing 827 unique miRNAs. Figure 2B demonstrates the experimental design. A total of 100 ng of total RNA was used per sample. Per the manufacturer’s instructions, specific DNA miR tags were ligated to the 3′ ends of sample miRNAs. Then, the samples were hybridized after removing excess tags with miR:tag-specific capture and reporter probes. Hybridization was carried out at 64 °C for 18 h, and the hybridized samples were loaded onto a streptavidin-coated nCounter Sprint cartridge. The cartridge was then read on the nCounter Sprint Profiler (Nanostring, Seattle, WA, USA), which counted individual fluorescent barcodes attached to miRNA targets.

miRNA target normalization was performed with the geometric mean of the top 100 miRNAs detected and background subtraction of 36 counts (the average of exogenous negative controls in the panel plus two standard deviations). Following normalization, all miRNAs with average counts of <5 within a sample group were excluded to ensure robust expression within the sample.

### 2.8. Data Analysis

Analysis of organoid viability data was performed using Prism 8 (GraphPad, Boston, MA, USA). miRNA analysis results were normalized using nSolver 4.0 (Nanostring) and analyzed within nSolver as well as Ingenuity Pathway Analysis software Version 1 (IPA, Qiagen). qPCR data were interpreted using the Thermo Fisher Digital Science online application (Thermo Fisher).

## 3. Results

### 3.1. Organoid Viability

**Figure 3:**
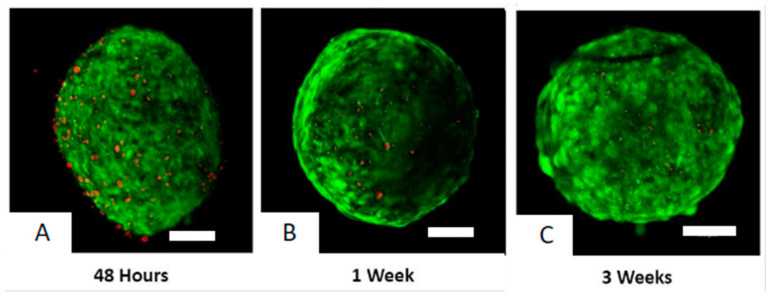
Organoid viability using Live-Dead Kit and confocal microscopy at 48 h (**A**), 1 week differentiation (**B**), and 3 week differentiation (**C**). (live cells: green, dead cells: red). White bars are 400 microns in length.

The viability of differentiating HTOs was assessed at formation and then weekly. Live-Dead confocal microscopy demonstrated excellent viability of HTOs at all time points with progressive loss of size due to less cell proliferation, cell differentiation, and organoid compaction (Figure 3).

### 3.2. Culture Characterization

**Figure 4:**
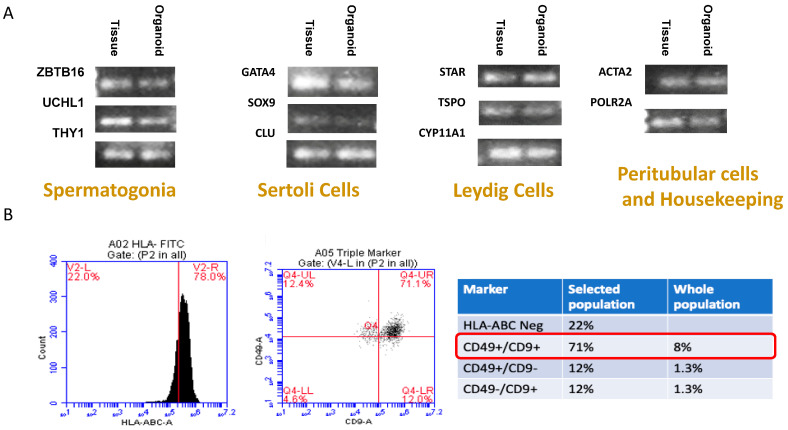
(**A**) RT q-PCR showed the presence of all significant testicular cell type in HTOs. (**B**) Flow cytometry quantified the enriched subpopulation of SSCs (HLA-ABC Negative/CD49 +/CD9+) in HTOs (Circled in red).

RT-qPCR confirmed the expression of cell markers indicating the presence of spermatogonia, Leydig cells, Sertoli cells, and peritubular cells in the long-standing spermatogonial stem cell culture. The results demonstrated comparable populations of cells following passage in culture to the whole testis tissue of subjects from which they were derived (Figure 4A).

Flow cytometry confirmed the presence of SSCs in formed HTOs.

SSCs are a subpopulation of spermatogonia cells that are HLA negative and CD49+/CD9+ (Figure 4B).

### 3.3. miRNA Expression Profiles

**Figure 5:**
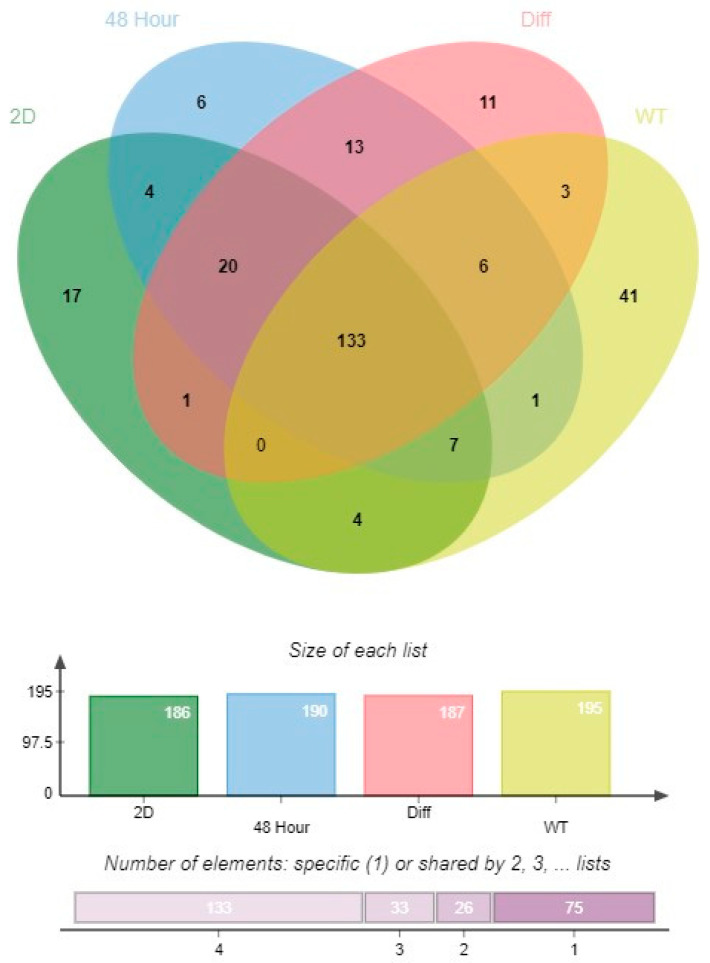
Venn Diagram of miRNA profiles. Comparison of the adult human testis (WT) represents in vivo condition, 2D SSC culture, and 3D HTO (before and after differentiation).

The samples of total RNA were analyzed for their miRNA content to compare the miRNA profiles of WT, 2D culture, and differentiated (3-week) 3D HTOs. One hundred ninety-five miRNAs met the criteria for expression in WT, 186 in 2D culture, 190 formed HTOs in 48 h, and 187 in differentiated organoids. Additionally, changes in miRNA expression between different stages (WT, 2D culture, 48 h formed HTOs, and differentiated HTOs) of testis cell culture were assessed using a Venn diagram generated by jvenn (Figure 5) [35]. In this comparison, 133 miRNAs were common across all conditions, and 41, 17, 6, and 11 miRNAs were unique in WT, 2D culture, 48 h formed HTOs, and differentiated HTOs, respectively.

When WT and differentiated HTOs were compared directly, 142 miRNAs were expressed in both conditions, while 53 were unique for WT and 45 to differentiated HTOs (Table 1). Of the commonly expressed miRNAs, 61 significantly differed between the two conditions (Table 1), though only two, miR-10b-5p and miR-21-5p, survived the application of false discovery rate (FDR) adjustment.

Additionally, we were interested in similarities in miRNA profiles, as the differentiated HTO is capable of spermatogenesis, and, in our belief, it is the closest in vitro approximation of the testis within our culture system. For this comparison, miRNAs that were not significantly different (*p* > 0.05) and with a factor change ratio between −1.25 and 1.25 were considered similar between the two conditions. Using this definition, 22 miRNAs were identical between WT tissue and differentiated HTOs (Table 2).

## 4. Discussion

**Figure 6:**
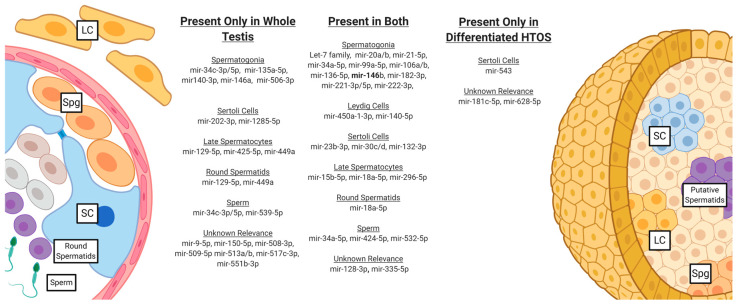
Comparison of miRNAs present in whole testis (WT) and differentiated HTOs.

miRNAs have emerged over the past two decades as critical post-transcriptional modulators of gene expression and key players in spermatogenesis. However, most of the studies evaluating the role of miRNAs in spermatogenesis have involved animal models and semen samples or focused on isolated cells from testis, and none have sought to assess changes in miRNA expression throughout the process of in vitro spermatogenesis. This study evaluated the miRNA expression profiles of progressively complex stages of testicular cell culture, culminating in a three-dimensional organoid model capable of meiotic differentiation. We compared these to the whole testis miRNA expression. Using the Nanostring Sprint Profiler system, we found 266 unique miRNAs expressed across the whole testis tissue and our culture system, with about half of these expressed across all sample groups. The whole testis had 41 unique miRNAs among the groups, compared to only 11 in differentiated HTOs. This suggests that while many miRNAs expressed in the whole testis are similarly found in our culture system, there remains a considerable variation from native tissue. Reviewing the available literature, we identified miRNAs with known biological relevance. Figure 6 demonstrates the cell types within the testis affected by miRNAs and compares the miRNAs expressed in the whole testis and differentiated HTOs. The miRNAs in both samples carry biologically relevant functions to the different functional cells within the testis, including spermatogonia, Leydig cells, Sertoli cells, and spermatids.

Among the miRNAs expressed in both native tissue and our system, most had functions on spermatogonial maintenance. The let-7 family of miRNAs has been shown to have roles in differentiation events in various tissues throughout the human body. The let-7 family, eight of which are expressed across all levels of this experiment, acts as a differentiation and antiproliferation factor in human spermatogonia, and its expression is increased in response to retinoic acid induction [36]. When compared between the whole testis tissue and differentiated HTOs, five of the eight let-7 miRNAs expressed in our samples had significantly different expressions between the two conditions; however, none met FDR. Mir-20 and mir-21-5p also play roles in SSCs maintenance, mir-20 (with mir-106a) via targeting STAT3 and mir-21-5p through regulating germ cell apoptosis [26,37]. Interestingly, mir-21-5p is upregulated over 10-fold in differentiated HTOs compared to the whole testis. In their 2011 paper on the mir-21 regulation of mouse SSCs, Niu et al. [26] linked mir-21 expression to the expression of ETV-5, a transcription factor and downstream target of GDNF signaling expressed in the testis in SSCs and Sertoli cells. They additionally found that inhibiting mir-21 increased apoptosis in SSC-enriched germ cell cultures and that mir-21 may be critical to early steps in spermatogenesis through apoptosis regulation. The significant difference in mir-21 expression between our culture system and native tissue may result from laboratory manipulation, as ETV-5 is expressed in response to signaling from GDNF, which is found ubiquitously in the media of our culture system. Mir-99, -136, -182, -221-3p, and -222-3p are expressed in both our system and the whole testis tissue and play similar roles in regulating genes contributing to apoptosis, germ cell maintenance, and retinoic acid-induced differentiation. Some miRNAs with relevant functions are found only in the whole testis tissue, including mir-135a, which in studies of cryptorchid testes has been shown to contribute to SSC maintenance through FOXO1 modulation [38]. Another mir-140-3p has been shown to have reduced expression during the transformation of type B spermatogonia to primary spermatocytes [39]. As these cells differ in their location within the germ cell niche, their lack of expression in our culture system could denote a function for mir-140-3p in localizing primordial germ cells to the basement membrane of the seminiferous tubule.

Beyond spermatogonia, we also observed the presence of miRNAs in HTOs with functions relevant to developing germ cells, including spermatocytes, round spermatids, and sperm. Mir-15b-5p, -18a-5p, and 296-5p were expressed in both the whole testis and HTOs and have been shown to play roles ranging from cell proliferation to cell apoptosis. Mir-15b, specifically, plays a role in the maturation of spermatocytes through the regulation of p53 and caspase9, suggesting it also regulates cell apoptosis [40]. Mir-18a is a member of the mir-17-92 cluster, which, as stated previously, is heavily involved in spermatogenesis, from maintaining spermatogonia to meiotic differentiation. In the whole testis, we identified several miRNAs with functions relevant to processes in late spermatocytes, early spermatids, and sperm, which are not specified in our organoid system. miR-129-5p is present in late spermatocytes and early spermatids and has a hypothesized function in the heat stress response pathway [41]; its absence in our organoid system may be explained by the constant thermal conditions under which it is held, compared to conditions which the native testis experiences. Mirs-34c and -449 carry essential, redundant functions to sperm development, as demonstrated by double knock-out mice experiencing infertility due to severe spermatogenic dysfunction in one study [42]. In the same study, the injection of these sperm into wild-type oocytes led to a block at the pro-nucleus to zygote transition. However, the injection of spermatids led to normal development. While these miRNAs are not present in our organoid system, this study shows that they might not be necessary for fertility, as technologies like ROSI can use the spermatids generated through organoid differentiation. miR-34 is famously known for its function in sperm tail development [25], so it is not found in our system, which lacks the intricate architecture of the seminiferous tubule.

Our study is not without limitations. This work is limited by the small number of subjects used to generate the tissue utilized in the study. Due to the cost of technology, samples were run as biological replicates of the three subjects. This likely led to the lack of significant differences in miRNA expression identified after applying the false discovery rate. An additional limitation encountered in our study is the lack of comparison of miRNA to target mRNA expression, which would have served to solidify the linkage between regulating miRNA and the final gene product. Future research in this sector should aim to characterize further the intricate function of miRNAs identified in this study on spermatogenesis, particularly within in vitro culture systems.

We anticipate several impacts of this study on the current understanding of the role of sncRNAs in human spermatogenesis. We have identified several miRNAs with reported biological function and confirmed their expression throughout several stages of in vitro culture. These data strengthen the current body of evidence for these miRNAs in spermatogenesis and provide new avenues for the study of miRNAs not yet described. Additionally, this study evaluates a known three-dimensional culture system which has been shown to be capable of spermatogenic differentiation and compares it to whole testis tissue, identifying miRNAs shared between the two that could play key roles in this process.

## 5. Conclusions

In the present study, we evaluated the changes in miRNA expression within a three-dimensional culture system capable of spermatogenesis compared to whole testis tissue. We identified a significant difference in miRNA expression between our culture system and the normal testis; however, some miRNAs were preserved. These data provide context for understanding the process of spermatogenesis both in vivo and in the laboratory setting and provide potential avenues for strengthening the latter process.

## Figures and Tables

**Table 1:** The differences between miRNA profiles of human whole testis (WT) and differentiated HTOs.

Micro RNA	Accession #	Count: Differentiated HTO	SD	Count: Whole Testis	SD	Ratio: Differentiated HTO vs. Whole Testis	*p* Value	FDR Adjusted *p* Value
hsa-miR-10b-5p	MIMAT0000254	1200.23	60.36	383.78	16.77	3.13	<0.001	0.02
hsa-miR-21-5p	MIMAT0000076	16,760.51	2262.3	1388.78	246.48	12.07	<0.001	0.04
hsa-miR-191-5p	MIMAT0000440	1616.98	87.59	829.35	57.15	1.95	<0.001	0.11
hsa-miR-4443	MIMAT0018961	137.95	6.13	36.62	3.53	3.77	<0.001	0.13
hsa-miR-34a-5p	MIMAT0000255	4227.95	968.56	514.26	90.02	8.22	<0.001	0.13
hsa-miR-149-5p	MIMAT0000450	14.41	2.1	44.99	5.38	−3.12	0.001	0.15
hsa-miR-100-5p	MIMAT0000098	7127.82	1549.68	599.72	53.35	11.89	0.001	0.15
hsa-miR-20a-5p+hsa-miR-20b-5p	MIMAT0000075	185.38	18.53	756.22	22.04	−4.08	0.001	0.18
hsa-miR-4516	MIMAT0019053	1319.43	936.22	16.81	10.91	78.49	0.001	0.18
hsa-miR-199a-3p+hsa-miR-199b-3p	MIMAT0000232	16,327.05	3656.88	3713.74	744.86	4.4	0.001	0.21
hsa-miR-132-3p	MIMAT0000426	1383.47	239.95	140.24	42.71	9.86	0.001	0.22
hsa-miR-199b-5p	MIMAT0000263	3168.82	570.56	277.43	88.37	11.42	0.001	0.27
hsa-miR-324-5p	MIMAT0000761	127.1	20.66	47.54	7.2	2.67	0.002	0.27
hsa-miR-126-3p	MIMAT0000445	26.69	9.5	1407.69	1063.11	−52.75	0.002	0.29
hsa-miR-656-3p	MIMAT0003332	112.54	17.51	17.88	5.52	6.29	0.002	0.29
hsa-miR-574-3p	MIMAT0003239	232.68	50.37	70.34	14.97	3.31	0.002	0.29
hsa-miR-1915-3p	MIMAT0007892	326.46	154.96	10	6.76	32.65	0.002	0.29
hsa-miR-28-5p	MIMAT0000085	243.28	34.32	107.6	14.82	2.26	0.002	0.29
hsa-miR-299-5p	MIMAT0002890	255.86	47.42	88.84	11.52	2.88	0.003	0.34
hsa-miR-494-3p	MIMAT0002816	85.63	19.8	21.8	2.89	3.93	0.003	0.35
hsa-miR-374a-5p	MIMAT0000727	3429.96	329.54	788.5	178.22	4.35	0.003	0.36
hsa-miR-454-3p	MIMAT0003885	36.02	7.62	11.21	1.15	3.21	0.003	0.38
hsa-miR-19a-3p	MIMAT0000073	112.43	9.37	305.66	58.44	−2.72	0.004	0.44
hsa-miR-337-3p	MIMAT0000754	86.29	28.61	20.35	4.91	4.24	0.004	0.44
hsa-let-7d-5p	MIMAT0000065	1133.24	190.53	545.61	76.95	2.08	0.004	0.48
hsa-miR-24-3p	MIMAT0000080	347.75	90.56	90.88	27.43	3.83	0.005	0.5
hsa-miR-497-5p	MIMAT0002820	165.53	74.19	1130.1	366.99	−6.83	0.007	0.64
hsa-let-7a-5p	MIMAT0000062	28,438.82	4218.04	6958.53	2037.02	4.09	0.007	0.66
hsa-miR-340-5p	MIMAT0004692	39.73	1.37	60.28	5.17	−1.52	0.007	0.66
hsa-miR-382-5p	MIMAT0000737	410	52.18	69.43	24.2	5.91	0.007	0.67
hsa-miR-199a-5p	MIMAT0000231	7133.99	2265.32	1925.62	241.68	3.7	0.008	0.68
hsa-miR-4454+hsa-miR-7975	MIMAT0018976	7363.49	3535.37	60,798.29	31,832.32	−8.26	0.008	0.7
hsa-miR-195-5p	MIMAT0000461	73.33	32.71	316.97	90.77	−4.32	0.008	0.7
hsa-miR-29b-3p	MIMAT0000100	10,994.69	3044.41	3690.16	844.93	2.98	0.008	0.7
hsa-miR-411-5p	MIMAT0003329	252.66	50.11	71.26	23.61	3.55	0.010	0.78
hsa-miR-181b-5p+hsa-miR-181d-5p	MIMAT0000257	128.86	18.38	13.64	6.68	9.45	0.012	0.92
hsa-miR-214-3p	MIMAT0000271	440.69	182.59	85.74	7.23	5.14	0.013	0.93
hsa-miR-140-5p	MIMAT0000431	219.29	31.37	134.62	20.04	1.63	0.014	0.98
hsa-let-7e-5p	MIMAT0000066	1530.35	514.82	368.91	12.64	4.15	0.014	0.98
hsa-miR-222-3p	MIMAT0000279	1291.16	137.36	99.33	72.13	13	0.016	1
hsa-miR-125b-5p	MIMAT0000423	55,042.04	2371.08	14,946.62	4495.45	3.68	0.018	1
hsa-let-7i-5p	MIMAT0000415	3609.14	1781.31	270.52	213.17	13.34	0.020	1
hsa-miR-423-3p	MIMAT0001340	75.8	16.61	36.53	2.02	2.07	0.025	1
hsa-miR-151a-5p	MIMAT0004697	111.64	9.04	61.83	12.31	1.81	0.027	1
hsa-miR-93-5p	MIMAT0000093	475.06	115.72	254.65	52.54	1.87	0.027	1
hsa-miR-181a-5p	MIMAT0000256	3259.44	852.17	1104.04	553.64	2.95	0.029	1
hsa-miR-15a-5p	MIMAT0000068	1994.78	916.55	678.24	116.92	2.94	0.030	1
hsa-miR-19b-3p	MIMAT0000074	322.42	111.7	929.39	74.56	−2.88	0.030	1
hsa-miR-25-3p	MIMAT0000081	967.8	180.1	546.29	19	1.77	0.030	1
hsa-miR-155-5p	MIMAT0000646	156.89	54.94	30.87	18.87	5.08	0.032	1
hsa-miR-337-5p	MIMAT0004695	132.27	28.81	32.91	21.57	4.02	0.032	1
hsa-miR-379-5p	MIMAT0000733	579.51	97.5	193.29	93.59	3	0.032	1
hsa-miR-125a-5p	MIMAT0000443	1721.59	105.77	572.19	206.74	3.01	0.036	1
hsa-miR-362-3p	MIMAT0004683	196.94	89.63	56.66	2.75	3.48	0.037	1
hsa-miR-409-3p	MIMAT0001639	287.22	68.71	22	20.61	13.06	0.040	1
hsa-miR-23a-3p	MIMAT0000078	9417.15	422.13	3390.98	1375.87	2.78	0.040	1
hsa-miR-130a-3p	MIMAT0000425	2008.96	43.27	1103.44	234.44	1.82	0.041	1
hsa-miR-30a-3p	MIMAT0000088	100.16	21.95	42.26	16.07	2.37	0.044	1
hsa-let-7g-5p	MIMAT0000414	3305.55	290.45	1874.25	434.71	1.76	0.045	1
hsa-miR-107	MIMAT0000104	236.27	24.03	110.42	34.11	2.14	0.046	1
hsa-miR-500a-5p+hsa-miR-501-5p	MIMAT0004773	113.98	74.09	33.9	16.41	3.36	0.049	1

**Table 2:** The similarities between miRNA profiles of human WT and differentiated HTOs.

Micro RNA	Accession #	Count: Differentiated HTO	SD	Count: Whole Testis	SD	Ratio: Differentiated HTO vs. Whole Testis	*p* Value	FDR Adjusted *p* Value
hsa-miR-30e-5p	MIMAT0000692	158.98	26.11	197.36	112.12	−1.24	0.533	1
hsa-miR-3144-3p	MIMAT0015015	35.92	17.54	44.6	48.22	−1.24	0.726	1
hsa-miR-18a-5p	MIMAT0000072	38.09	6.77	46.93	6.5	−1.23	0.190	1
hsa-miR-450a-5p	MIMAT0001545	1022.21	531.67	1243.36	268.27	−1.22	0.585	1
hsa-miR-335-5p	MIMAT0000765	31.79	19.97	37.85	6.93	−1.19	0.655	1
hsa-miR-1260a	MIMAT0005911	41.89	25.67	48.15	5.83	−1.15	0.719	1
hsa-miR-29c-3p	MIMAT0000681	1039.99	184.25	1188.48	265.07	−1.14	0.446	1
hsa-miR-612	MIMAT0003280	168.68	61.15	193.04	235.53	−1.14	0.829	1
hsa-miR-148a-3p	MIMAT0000243	906.63	173.39	970.51	195.8	−1.07	0.691	1
hsa-miR-128-3p	MIMAT0000424	34.62	16.66	36.77	5.55	−1.06	0.843	1
hsa-miR-186-5p	MIMAT0000456	117.72	32.73	117.35	18.55	1	0.987	1
hsa-miR-106b-5p	MIMAT0000680	93.14	59.01	93.02	26.47	1	0.997	1
hsa-miR-450a-1-3p	MIMAT0022700	13.42	5.57	13.4	11.42	1	0.998	1
hsa-miR-30d-5p	MIMAT0000245	190.08	46.72	189.11	48.36	1.01	0.981	1
hsa-miR-23b-3p	MIMAT0000418	499.24	138.72	488.41	33.92	1.02	0.905	1
hsa-let-7c-5p	MIMAT0000064	2970.6	648.38	2890.79	360.98	1.03	0.862	1
hsa-miR-218-5p	MIMAT0000275	84.49	38.09	80.06	37.7	1.06	0.889	1
hsa-miR-664a-3p	MIMAT0005949	69.42	17.13	64.64	32.73	1.07	0.829	1
hsa-miR-548ar-5p	MIMAT0022265	43.61	2.38	40.82	40.42	1.07	0.911	1
hsa-miR-30e-3p	MIMAT0000693	94.53	31.32	87.2	8.68	1.08	0.737	1
hsa-miR-361-3p	MIMAT0004682	101.28	16.6	93.93	48.41	1.08	0.812	1
hsa-miR-590-5p	MIMAT0003258	30.92	12.26	25.75	9.11	1.2	0.573	1

## Data Availability

The original contributions presented in the study are included in the article, further inquiries can be directed to the corresponding author/s.
